# Message reactance as a mediator of effects of alcohol and cancer risk messages

**DOI:** 10.1186/s12889-026-26769-8

**Published:** 2026-03-07

**Authors:** Jacob A. Rohde, Jackelyn B. Payne, Carlos O. Garrido, Emma Jesch, Rebecca A. Ferrer, Richard P. Moser, David Berrigan, William M. P. Klein, Paul K.J. Han

**Affiliations:** 1 Department of Allied Health Sciences, University of Connecticut, 358 Mansfield Rd, U-1101, Storrs, CT 06269 USA; 2 Cancer Prevention Fellowship Program, Behavioral Research Program, Division of Cancer Control and Population Sciences, National Cancer Institute, Bethesda, MD 20850 USA; 3 Division of Integrative Biological and Behavioral Sciences, National Institute on Minority Health and Health Disparities, Bethesda, MD 20892 USA; 4 Behavioral Research Program, Division of Cancer Control and Population Sciences, National Cancer Institute, Bethesda, MD 20850 USA

**Keywords:** Alcohol consumption, cancer prevention, risk messages, message reactance

## Abstract

**Background:**

This study examined whether message reactance mediates the cognitive effects of messages about the causal relationship between alcohol and cancer.

**Methods:**

We randomly assigned US adults (*N* = 799) recruited from a commercial online research panel to receive one of two types of messages about the alcohol-cancer link: (1) high-certainty (e.g., “Drinking alcohol causes cancer”), and (2) low-certainty, employing the modal verb “may” (e.g., “Drinking alcohol may cause cancer”). We evaluated whether message reactance mediated the effects of message certainty vs. uncertainty on (1) perceived causal certainty about the alcohol-cancer link, and (2) perceived alcohol-related cancer risk. We also explored whether heavy vs. non-heavy alcohol consumption moderated these effects.

**Results:**

Message reactance mediated the effects of high-certainty (vs. low) messages on perceived causal certainty (direct: *b*=0.54; indirect: *b*=-0.11; both *p*s<0.001) and alcohol-related cancer risk (direct: *b*=0.37; indirect: *b*=-0.13; both *p*s<0.001)—suppressing the positive effects of expressed certainty on both outcomes. The suppressive effects of message reactance on perceived causal certainty were stronger for participants reporting heavy (*b*=-0.50) vs. non-heavy alcohol consumption (*b*=-0.35; *p*=.034). Conditional indirect effects for the perceived causal certainty model only remained significant for participants with non-heavy alcohol consumption (*b*=-0.10; *p*=.004).

**Conclusions:**

Effects of high-certainty (vs. low) messages were negatively mediated by message reactance and moderated by alcohol consumption. Findings suggest that although high-certainty risk messages may increase cancer risk perceptions, their effect is attenuated by inciting negative reactance to the messages, particularly for those who consume a heavy amount of alcohol. These mixed effects should be considered in future messaging initiatives aimed at communicating the alcohol-cancer relationship to the public.

## Introduction

Consuming alcoholic beverages increases the risk for several malignancies, including cancers of the breast, liver, colon, esophagus, as well as head and neck [1–3]. Estimates suggest only between one-third and one-half of US adults are aware that drinking alcohol can cause cancer [4, 5]. Raising public awareness of the alcohol-cancer relationship is critical to ensure that individuals have the information to make an informed decision about whether or how much to drink alcohol. However, the most effective strategies for raising such awareness remain to be determined [6, 7]. One potential awareness strategy consists of alcohol-cancer warning messages, which could be disseminated through various communication channels (e.g., public service announcements, beverage warning labels). However, such messages can differ in numerous ways, including their substantive content and the specific language used to communicate risk, and the optimal design of these messages and their effects on recipients remains to be determined. Our prior experiment explored these message differences [17]. Specifically, the study found that varying the type of causal language in alcohol-cancer risk messages (e.g., “Drinking alcohol [*causes* vs. *is linked to*]* cancer*”) had no effect on key outcomes, including behavioral intentions; however, adding modal verb modifiers to those same messages (e.g., “Drinking alcohol [*may cause* vs. *causes*] cancer”) decreased perceptions of alcohol-cancer risk and causal certainty, yet also lowered negative message reactance. The current study sought to build on the main effects observed in Payne et al. [17] by testing if reactance mediated the cognitive effects of risk messages communicating the causal relationship between alcohol and cancer.

### Literature review

One important question in the design of risk messages is how to describe the causal relationship between the risk factor and the health outcome. Messages using strong causal language may affect people’s causal beliefs, an acknowledged determinant of health behaviors. For example, in the common-sense model of self-regulation of health and illness, causal beliefs influence the extent to which people engage in illness-specific, health-protective behaviors [8, 9]. As such, messages that reinforce a belief that alcohol causes cancer may be more effective in encouraging people to avoid or reduce consumption.

Causal language may also influence risk perceptions by expressing certainty (vs. uncertainty) about the occurrence or likelihood of a given outcome. For example, stating that “alcohol *causes* cancer” conveys a high degree of certainty that cancer will eventually result from drinking alcohol, compared to similar language that tempers the causal link—e.g., “alcohol *may* cause cancer.” The addition of a modal verb (e.g., lexical expressions, such as “may” or “might,” that express tentativeness or possibility about subsequent claims in a sentence) to causal messages adds uncertainty about the outcome being discussed [10–12].

The potential effects of differing causal language on responses to risk messages raises the need to understand how such language should be incorporated in designing messages to communicate the alcohol-cancer relationship. On the one hand, health communicators interested in increasing cancer risk perceptions may wish to use high-certainty causal language (e.g., “alcohol causes cancer”). However, scientific uncertainty about alcohol-associated cancer risks, variation in individuals’ cancer susceptibility and risk-reducing behaviors, and the inherent unpredictability of health outcomes may all justify lower-certainty language (e.g., “alcohol *may* cause cancer) [13]. The appropriateness of modifying causal language in alcohol-cancer risk messages is both a normative and empirical question that depends on the goals of communication (e.g., to inform or warn the public vs. to promote behavior change) and on the extent to which a given message conveys the strength of the causal relationship to message recipients.

Available empirical evidence suggests that lower-certainty health messages that incorporated modal language produced diminishing effects on risk perceptions and behaviors. In an experimental study of brief alcohol warning messages focused on multiple health risks (e.g., liver disease), Hall and colleagues (2019) compared the effects of four linguistic strategies for describing the causal influence of alcohol: “causes,” “contributes to,” “can contribute to,” and “may contribute to” [14]. The message that used the most certain language—“Drinking alcohol *causes* liver disease”–was rated by participants as most discouraging them from wanting to drink alcohol. In contrast, the less-certain message stating, “Drinking alcohol *may contribute to* liver disease” was rated as least discouraging them from wanting to drink alcohol. These findings were corroborated by a similar alcohol risk message experiment [15]. Another study by Ma and Ma (2022) integrated multiple modal words (e.g., may, could, probably) in long-form narrative messages communicating the cancer risk associated with alcohol [16]. This study found that use of low-certainty language decreased intentions to reduce or stop drinking. Moreover, this effect was mediated by increased perceptions of message tentativeness and decreased cancer risk perceptions, indicating that mechanisms, such as receptivity, play a key role in understanding intended message effects.

These studies suggest that low-certainty (vs. high) language might negatively affect public perceptions of alcohol-related health risks [15, 16]. However, many important factors in this association remain to be determined. For example, our prior study [17] found that high- vs. low-certainty causal language increased both cancer risk perceptions and negative message reactance—a negative motivational state triggered when one’s freedom is eliminated or threatened [18, 19]. Although our prior study did not investigate how these effects might relate to each other, reactance could be an important mediator of the effects of high-certainty causal language on perceptions of the cancer risk associated with alcohol. To the extent that high-certainty causal language implies fundamental limits to one’s agency and freedom, use of such language may increase reactance to health risk messages.

In turn, reactance may diminish the psychological effects of these messages, which has been shown in studies examining pictorial cigarette warning messages and less consistently in some studies examining alcohol risk messages [12, 16, 17, 20–23]. For example, a recent experiment by Grummon and colleagues tested the effects of messages communicating different alcohol risks (e.g., liver disease, dementia, various cancer types) and found reactance to be high for messages communicating the link between alcohol and most types of cancer (or generic cancer), particularly when compared to a control message about recycling alcoholic beverage containers [15]. Notably, our prior experiment also showed a main effect of message condition on reactance, such that exposure to high-certainty messages increased reactance [17]. Because reactance is a message receptivity outcome that typically manifests soon after message exposure [24], reactance should mediate the effects of alcohol–cancer risk messages on other downstream outcomes, such as risk perceptions.

### Study aims

The current study extended our prior work [17] by testing the extent to which message reactance mediated the effects of high- vs. low-certainty causal language in messages about the relationship between alcohol and cancer. We hypothesized that the effects of high- vs. low-certainty messages (e.g., “drinking alcohol causes cancer” vs. “drinking alcohol *may* cause cancer”) on perceptions of causal certainty and cancer risk would be mediated by reactance—specifically, that high-certainty messages would increase perceived causal certainty and cancer risk—but that this effect would be suppressed, or negatively mediated, by increasing reactance.

In addition, we explored a potential moderating effect of personal alcohol consumption on these effects. Research on defensive processing and motivated reasoning suggests that frequency of alcohol consumption may affect individuals’ receptivity to alcohol-cancer risk messages [25]. Individuals who drink alcohol regularly, for example, may perceive such messages as especially threatening to their identity, compared to individuals who drink less or who abstain from drinking alcohol. Thus, alcohol consumption may moderate the negative effect of high-certainty causal language on perceptions of causal certainty and cancer risk, with heavy levels expected to promote a strong negative effect.

## Methods

### Study design and participants

The current study was a between-subjects experiment conducted among a convenience sample (*N* = 799) of US adults, aged 18 years and older. Study participants were recruited from an opt-in online research panel maintained by the commercial vendor Prolific, which invites panel members to participate in studies for monetary incentives. Participants were recruited and randomized in February 2023 to a 4 × 2 factorial experiment that manipulated both causal language and modal language. Participants were compensated $3.00 USD for completing a survey that required approximately 15 min (~$12.00 USD/hour). All participants provided informed consent prior to completing the survey.

The first experimental factor, *causal language*, consisted of four different descriptions of the association between alcohol and cancer: (1) Drinking alcohol *causes* cancer, (2) Drinking alcohol *contributes to the risk* of cancer, (3) Drinking alcohol *increases the risk* of cancer, and (4) Drinking alcohol *is linked* to cancer. The second experimental factor, *modal language*, consisted of either including or omitting the word “may” to each of the causal language message variants describing the association between alcohol and cancer (e.g., Drinking alcohol [*may*] cause cancer), which resulted in two broad message groups: high-certainty (modal language absent) (*n* = 407) and low-certainty (modal language present) (*n* = 392). The current study focused on comparing these two groups (collapsing across the four different causal language conditions constituting the first factor), given our objective of evaluating the effects of expressed certainty vs. uncertainty via the inclusion of modal language modifiers in the risk messages. The study’s main analysis comparing outcomes of the four different causal messages comprising the first experimental factor is reported elsewhere [17]. The protocol was deemed exempt from review by the National Institutes of Health Institutional Review Board (#001139).

### Measures

*Perceived causal certainty about the relationship between alcohol and cancer.* Perceived causal certainty was measured with a 2-item scale developed for this study, with stems based on prior items from health surveys [26]: “Based on this message…” (1) how certain is the link between alcohol and cancer?, and (2) how strong is the link between alcohol and cancer? Responses to this item were assessed on the following 5-point scale: “not at all” (1), “a little” (2), “somewhat” (3), “quite a bit” (4), and “very much” (5). Items were averaged together to create a single composite measure (*α* = 0.94).

*Perceived alcohol-related cancer risk.* Perceived risk of cancer due to alcohol consumption was measured using a single item, also adapted from prior health behavior research. “Based on this message how likely do you think it is that alcohol causes cancer?” Responses were assessed on the same scale as perceived causal certainty about the relationship between alcohol and cancer outcome.

*Message reactance.* Message reactance was assessed with a brief 3-item scale used in prior research [20]. Participants responded to the following statements: (1) “This message is trying to manipulate me,” (2) “The health effect in this message is overblown,” and (3) “This message annoys me.” Responses to reactance were assessed on 5-point scales ranging from “strongly disagree” (1) to “strongly agree” (5). Items were averaged together to create a single composite measure (*α* = 0.83).

*Alcohol consumption.* Estimated lifetime alcohol consumption was first assessed by asking participants if they had ever had a drink of any alcoholic beverage in their lifetime. Those individuals who responded yes were asked how many drinks they consume in an average week, with response options ranging in single intervals from 0 to 15 or more than 15 drinks. We also assessed past 30-day binge drinking, which we defined as consuming more than 5 drinks on a single occasion for males or 4 drinks for females. Response options to the binge drink question included “never,” “1 to 2 times,” “3 to 5 times,” “6 to 10 times,” and “11 or more times.” Participants were shown a visual aid displaying the amount of liquid constituting one drink for different types of alcoholic beverages when answering these items. We then coded participants into one of two alcohol consumption categories: (1) non-heavy alcohol consumption; and (2) heavy alcohol consumption—defined as either consuming more than 14 (men) or 7 (women) drinks per week or reporting at least one binge drinking episode in the past 30 days. These questions and categories are in accordance with definitions from the National Institute on Alcohol Abuse and Alcoholism [27].

*Sociodemographic characteristics*. We collected participant reported age (numeric), sex (male, female, prefer not to answer), educational attainment, Hispanic ethnicity, race, sexual orientation, household income, and past cancer history (see Payne et al. for an overview of the sociodemographic characteristics of the sample for the current study) [17].

### Data analysis

We used the Hayes (2018) PROCESS model 4 approach to separately test whether message reactance (*M*) mediated the association between the experimental message condition (high-certainty vs. low-certainty[reference]; *X*) and the two study outcomes: perceived causal certainty about the alcohol-cancer relationship (*Y*_*1*_) and perceived alcohol-related cancer risk (*Y*_*2*_). Indirect effects were calculated by multiplying the *X* → *M* (denoted as *b*_*a*_) and *M* → *Y* (*b*_*b*_) path estimates, and the direct effect was presented by the *X* → *Y* (*b*_*c'*_) estimates. Total effects were calculated by the sum of direct and indirect effects. Each model used a bootstrap method with 1,000 repeated simulations to estimate model variance and adjusted for participant age, sex, race, education status, household income, sexual orientation, and heavy alcohol consumption.

Next, we separately computed two moderated-mediation models, one for each of our primary outcomes. We built on the mediation model structure described above, but stratified all mediation paths (*X* → *Y*, *X* → *M*, and *M* → *Y*) by heavy vs. non-heavy alcohol consumption status. We then interpreted simple slope estimates and conditional indirect effects (*b*_a_*b_*b*_) of *X* on *Y* mediated through *M*) for any path that posited a significant interaction term. Both models used a bootstrap method with 1,000 repeated simulations to estimate variance. Models adjusted for participant age, sex, race, education status, household income, and sexual orientation. All analyses were conducted in R (version 4.2.2). Main effects of the high- vs. low-certainty message condition on study outcomes are reported in a separate publication [17]. Broadly, results from that analysis found that exposure to high-certainty (vs. low) messages increased perceived causal certainty, perceived alcohol-related cancer risk, and negative message reactance outcomes.

## Results

### Mediating effects of message reactance

**Fig. 1:**
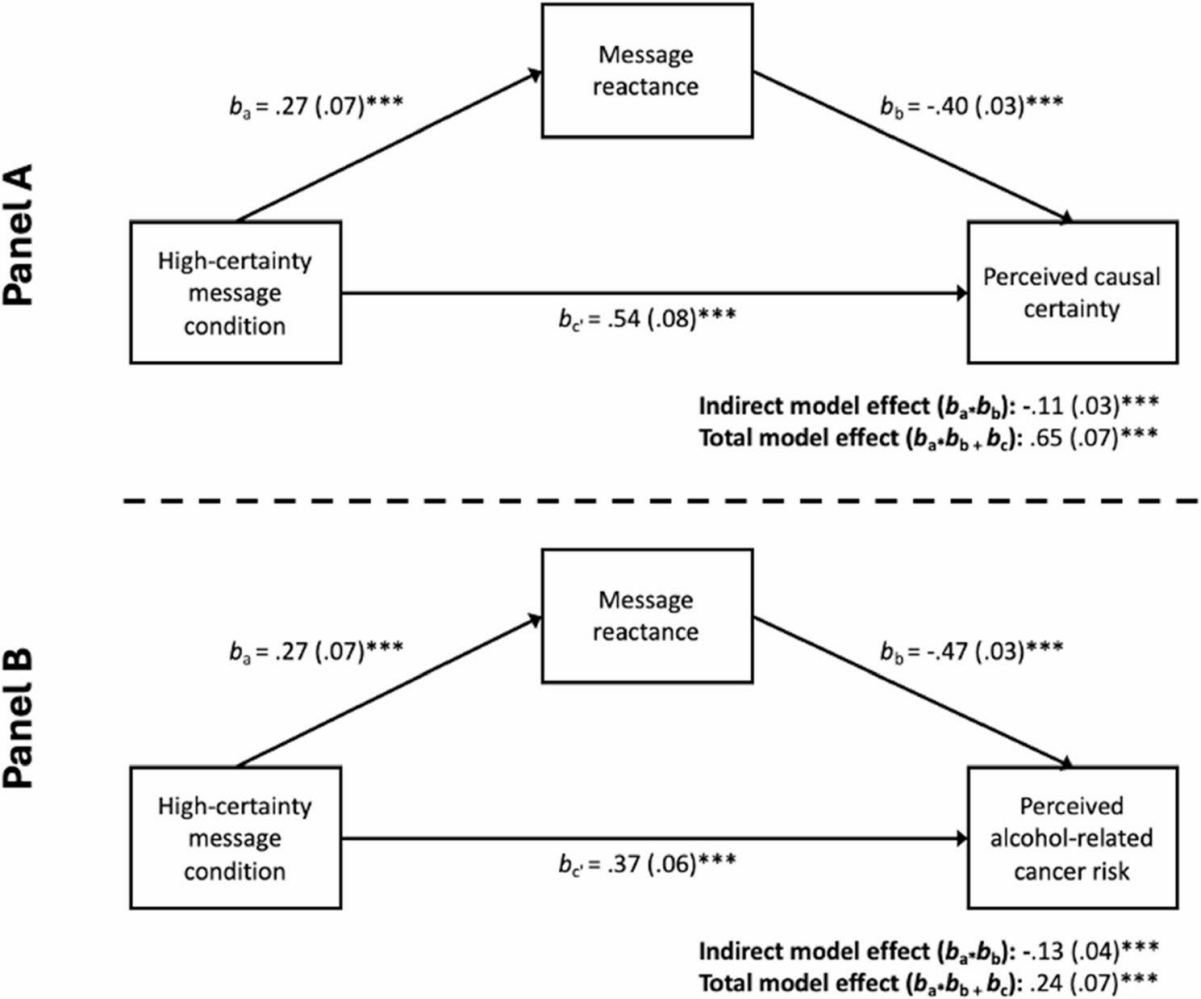
Effects of the high-certainty (vs. low) message condition on perceived causal certainty about the relationship between alcohol and cancer (Panel **A**) and perceived alcohol-related cancer risk (Panel** B**) mediated through message reactance; path estimates are unstandardized beta coefficients; values in parentheses are standard errors

As predicted, message reactance mediated the effect of high- vs. low-certainty language on perceived causal certainty about the relationship between alcohol and cancer (Figure 1). The high-certainty (vs. low) condition was positively associated with message reactance (*b*_*a*_=0.27, *p*<.001), and message reactance was negatively associated with perceived causal certainty (*b*_*b*_=-0.40, *p*<.001). The indirect mediation path was significant and negative, indicating that message reactance suppressed the effect of high-certainty messages (vs. low) on perceived causal certainty (indirect effect: *b*_a*_b_b_ =-0.11; direct effect: *b*_*c’*_=0.54; both *ps*<0.001). The pattern of mediation effects for the perceived alcohol-related cancer risk outcome was the same (indirect effect: *b*_a*_b_b_=-0.13; direct effect: *b*_*c’*_=0.37; both *ps*<0.001).

### Moderating effects of alcohol consumption on the mediation models

Most participants (71%; *n* = 565) self-reported non-heavy alcohol consumption (vs. heavy consumption: 29%; *n* = 234). The results indicated a significant moderating effect of alcohol consumption on the direct effect of message condition on perceived causal certainty (*X* → *Y*); *F*(1, 782) = 9.76, *p*=.002 (Table 1). Conditional slopes indicated that the positive effect of the high-certainty (vs. low) message condition was weaker for individuals who self-reported heavy (*b*=0.31, *p*<.001) vs. non-heavy alcohol consumption (*b*=0.80, *p*<.001).

**Table 1:** Conditional effects of high-certainty (vs. low) risk messages on perceived causal certainty moderated by alcohol consumption

Model path effects	Alcohol consumption	b (SE)	Interaction *p*
Direct c’ (X→Y)	Non-heavy	0.80 (0.08) ***	0.002**
	Heavy	0.31 (0.13)*	
Path a (X→M)	Non-heavy	0.27 (0.09)**	0.949
	Heavy	0.26 (0.14)	
Path b (M→Y)	Non-heavy	− 0.35 (0.04)***	0.034*
	Heavy	− 0.50 (0.06) ***	
Indirect (X→M→Y)	Non-heavy	− 0.10 (0.03)**	N/A
	Heavy	− 0.13 (0.07)	

Note. *b*=unstandardized beta coefficients

*SE *Standard error

*=*p*<.05; **=*p*<.01; ***=*p*<.001

**Fig. 2:**
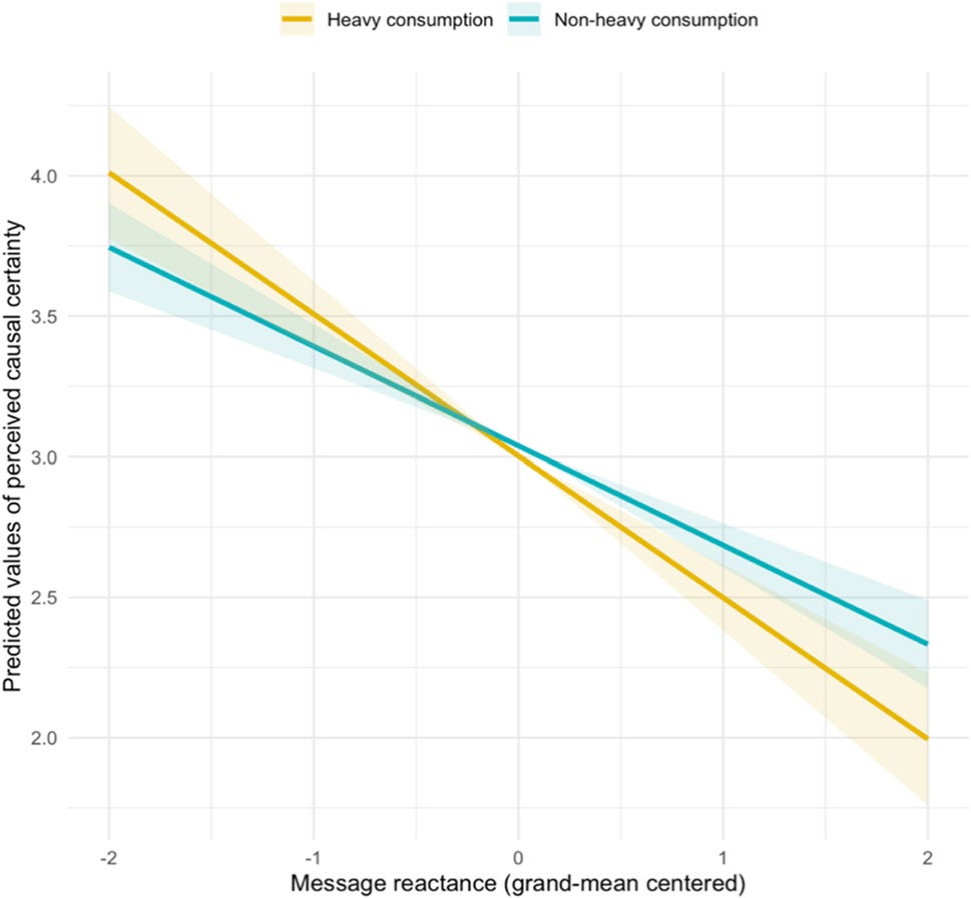
Conditional slopes of message reactance on perceived causal certainty about the relationship between alcohol and cancer stratified by alcohol consumption

The effect of message reactance on perceived causal certainty about the relationship between alcohol and cancer (*M* → *Y* path) was also moderated by alcohol consumption; *F*(1, 784) = 4.49, *p*=.034 (Table 1). Specifically, the negative effect of reactance on perceived causal certainty was stronger for individuals who self-reported heavy (*b*=-0.50, *p*<.001) vs. non-heavy alcohol consumption (*b*=-0.35, *p*<.001; Figure 2). Further, conditional indirect effects suggest that message reactance suppressed the positive effects of high-certainty message condition on perceived causal certainty, but only for individuals who self-reported non-heavy alcohol consumption (indirect effect: *b*_a*_b_b_=-0.10, *p*=.004); the same mediation path for heavy alcohol consumption was non-significant (indirect effect: *b*_a*_b_b_=-0.13, *p*=.067).

Alcohol consumption did not moderate the association between the message certainty condition and message reactance (M → Y) on the perceived causal certainty outcome. In addition, alcohol consumption did not moderate any indirect or direct path in the model testing message reactance as a mediator between the message certainty condition and the perceived alcohol-related cancer risk outcome.

## Discussion

This study tested the role of reactance in mediating important cognitive effects of messages about the causal relationship between alcohol and cancer. The results supported our predictions, indicating that reactance, in part, suppresses (i.e., negatively mediates) the effects of high- vs. low-certainty causal language on participants’ risk perceptions. Moreover, self-reported alcohol consumption moderated some of these effects, showing that the negative mediational effect of reactance remained persistent only for those who reported non-heavy (vs. heavy) alcohol consumption. These findings suggest that the causal language used in public messaging campaigns about the alcohol-cancer link may have mixed effects that also depend on the target audience.

Prior empirical work indicated that exposure to alcohol and cancer risk messages may elicit unintended negative reactance [15–17]. Building on this evidence, we hypothesized that reactance would *mediate* at least some effects of these messages on peoples’ alcohol-cancer risk perceptions. Indeed, participants in the current study exhibited more reactance when exposed to high- vs. low-certainty risk messages, which, in turn, mitigated effects on our two alcohol-cancer risk perception outcomes (an unintended consequence). Conversely, the heightened effects of low-certainty modal messages were positively mediated by message reactance. In other words, expressed causal certainty in alcohol-cancer risk messages have countervailing effects that could limit their impact; high-certainty (vs. low) messages may increase alcohol-cancer risk perceptions, yet simultaneously decrease such perceptions by heightening reactance.

Importantly, these mediation effects also differed by the level of alcohol consumption of message recipients. Findings indicated that the negative impact of reactance on perceived causal certainty about the alcohol-cancer link was stronger (i.e., more negative) among those who self-reported heavy alcohol consumption (vs. non-heavy). This finding may be attributable to reactance serving as a proxy for anger [28], which has been shown to decrease risk perceptions [29]. That is, people who consume a heavy amount of alcohol may experience heightened anger from risk messages about alcohol and cancer, likely explained by defensive processing. Such anger could, in turn, minimize their perceptions of risk. Alternatively, reactance may manifest individuals’ conscious or subconscious denigration of a message that is particularly threatening given their behavior. Although this study did not directly assess affective responses to the alcohol-cancer risk messages, items on the reactance scale do incorporate elements related to anger (e.g., “This message annoys me”).

It should be stated that our observed moderated-mediation was limited to the M→Y path, and only for the perceived causal certainty outcome (not the perceived alcohol-cancer risk outcome). Further, conditional indirect effects indicate that negative message reactance suppressed the effect of high-certainty (vs. low) messages on perceived causal certainty, but only among participants reporting non-heavy (vs. heavy) alcohol consumption. Although this finding appears counterintuitive, it is likely explained by observed differences in the conditional direct effects; after controlling for negative reactance, the association between the high-certainty message condition and perceived causal certainty was weaker among heavy vs. non-heavy consumers (difference in effect: −0.49, *p*=.002). In other words, high-certainty messages only exhibited a small direct effect on perceived causal certainty among heavy consumers, thus limiting the extent to which message reactance could meaningfully mediate that relationship. Additional research is needed to understand other factors that may influence our mediation models, particularly the effects of message condition on reactance (i.e., the X→Y path).

Taken together, our findings have practical implications for public health communication efforts. They suggest that use of high-certainty causal language in messages about the relationship between alcohol and cancer increases recognition of this relationship, and thus, may be the most effective way to warn the public about the risk. However, at some point, causal certainty in alcohol messages may undermine their effectiveness by triggering reactance. This possibility raises the need for messaging strategies that can reduce reactance in other ways, such as by affirming the values of people who drink alcohol (i.e., self-affirmation) or by avoiding disparaging/threatening language when communicating risks that may undermine people’s self-concept or sense of personal freedom [30, 31].

The study had some notable limitations. First, we utilized a convenience sample from a research panel. In addition, the experiment did not include a no-treatment control condition against which to compare varied levels of expressed certainty. Furthermore, our perceived alcohol-related cancer risk outcome was measured using a single item adapted for this study and has not been validated; however, it should be noted that the variability of the item was sufficient for its use in this study (i.e., approximately normally distributed, *M* = 2.75, *SD* = 1.07). Moreover, many of our variables assessed only immediate effects, rather than longer-term cognitive and behavioral outcomes (e.g., actual alcohol consumption behaviors). It should also be stated that our results are correlational and suggest, but do not establish, a causal mediational chain between message reactance and the effects of high- vs. low-certainty alcohol-cancer messages on the reported outcomes. Additional research addressing these limitations is needed to replicate and confirm our study findings.

## Conclusion

Risk messages that communicate the causal relationship between alcohol and cancer using high- vs. low-certainty language may increase perceptions of causal certainty and cancer risk. However, this effect is negatively mediated and attenuated by a countervailing effect on message reactance, which is greater among individuals with higher levels of alcohol consumption. Current and future public health communication initiatives may want to account for these nuances and consider tailoring messages according to individuals’ alcohol consumption history.

## Data Availability

The data used for the current study are available from the corresponding author on reasonable request.
